# Spontaneous representation of numerosity zero in a deep neural network for visual object recognition

**DOI:** 10.1016/j.isci.2021.103301

**Published:** 2021-10-15

**Authors:** Khaled Nasr, Andreas Nieder

**Affiliations:** 1 Animal Physiology Unit, Institute of Neurobiology, Auf der Morgenstelle 28, University of Tübingen, 72076 Tübingen, Germany

**Keywords:** Biological sciences, Neuroscience, Sensory neuroscience, Machine learning

## Abstract

Conceiving “nothing” as a numerical value zero is considered a sophisticated numerical capability that humans share with cognitively advanced animals. We demonstrate that representation of zero spontaneously emerges in a deep learning neural network without any number training. As a signature of numerical quantity representation, and similar to real neurons from animals, numerosity zero network units show maximum activity to empty sets and a gradual decrease in activity with increasing countable numerosities. This indicates that the network spontaneously ordered numerosity zero as the smallest numerical value along the number line. Removal of empty-set network units caused specific deficits in the network's judgment of numerosity zero, thus reflecting these units' functional relevance. These findings suggest that processing visual information is sufficient for a visual number sense that includes zero to emerge and explains why cognitively advanced animals with whom we share a nonverbal number system exhibit rudiments of numerosity zero.

## Highlights

• Zero representations spontaneously emerge in a neural network without training
• Network units are tuned not only to countable numerosity but also to zero
• Neural network orders zero as the smallest numerical value along the number line
• Zero units are functionally relevant for the network's discrimination performance

## Graphical abstract

**Figure:**
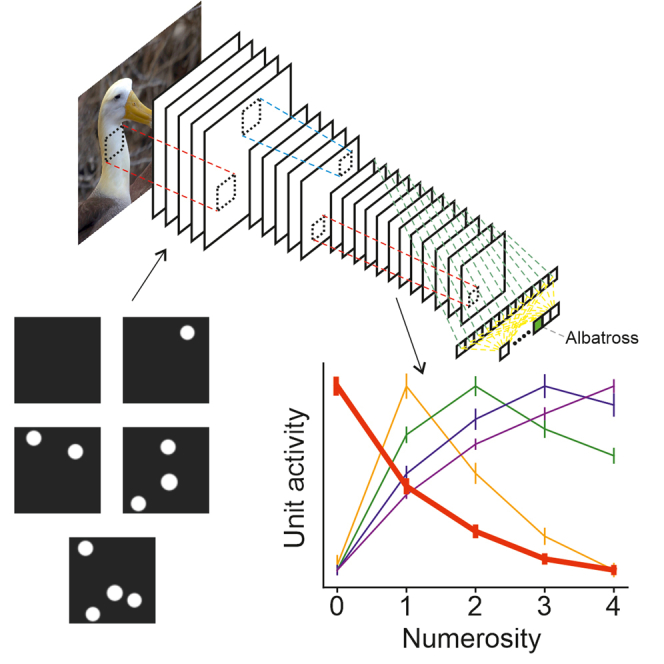

## Introduction

Humans and animals possess an intuitive capability to assess the number of items in a visual set, its numerosity. This “number sense” arises from the working of “number neurons” that are tuned to preferred numerosity (Nieder, 2016a; Kutter et al., 2018; Ditz and Nieder, 2020). Number neurons exist without prior number training in the brain of different vertebrate species (Viswanathan and Nieder, 2013; Wagener et al., 2018). Such neurons are thought to enable the inborn assessment of numerosity in newborn animals (Rugani et al., 2008) and human infants (Izard et al., 2009), as well as the automatic representation of numerical quantity in young children (Kersey and Cantlon, 2017) and adults (Park et al., 2016; Castaldi et al., 2016; Fornaciai and Park, 2018; Castaldi et al., 2020).

In an attempt to explain the spontaneous emergence of the number sense and its underlying number neurons in real brains, deep neural networks (DNNs) have been applied. In particular, biologically inspired hierarchical convolutional neural network (HCNN) that mimics the workings of the visual system proved successful (Stoianov and Zorzi, 2012; Nasr et al., 2019; Kim et al. 2021). Such HCNNs had previously achieved great success in computer vision applications (Krizhevsky et al., 2012; Simonyan and Zisserman, 2015) and in the modeling of visual shape processing (Yamins et al., 2014; Yamins and DiCarlo, 2016). These computational studies showed that network units tuned to visual numerosity, and with coding properties characteristic for real number neurons, spontaneously emerge in neural networks that were merely trained on visual object recognition (Nasr et al., 2019) or not trained on any task at all (Kim et al., 2021). This indicates that the spontaneous emergence of the number sense seems to be based on mechanisms inherent to the visual system.

However, one special numerical quantity, the empty set or numerosity zero, has remained computationally unexplored. The concept of zero is a late achievement in both human development (Merritt and Brannon, 2013) and history (Nieder, 2016b), probably because conceiving of empty sets (“nothing”) as a meaningful numerical category demands high-level abstraction. Surprisingly, even cognitively advanced animals, such as monkeys (Merritt et al., 2009; Ramirez-Cardenas et al., 2016), crows (Kirschhock et al., 2021), and honeybees (Howard et al., 2018), possess a primitive non-symbolic notion of zero. As a clear behavioral signature of a conception of numerosity zero, these species show a numerical distance effect with empty sets: they confuse numerosity 1 more often with the empty set than numerosity 2. The empty set is therefore not only “nothing” as opposed to “something,” but also represented together with countable numerosities as a numerical quantity on an ordered mental “number line” (Merritt et al., 2009; Merritt and Brannon, 2013; Nieder 2016b).

Recently, a neuronal code for numerosity zero has been discovered in the cerebral cortex of monkeys (Okuyama et al., 2015; Ramirez-Cardenas et al., 2016; Ramirez-Cardenas and Nieder, 2019) and the telencephalic pallium of crows (Kirschhock et al., 2021). Electrophysiological recordings in these behaving animals showed that single neurons responded to empty sets and were tuned to preferred numerosity zero (Okuyama et al., 2015; Ramirez-Cardenas et al., 2016; Kirschhock et al., 2021). Both in mental and neuronal representations, the numerosity zero is placed next to, and overlapped with, the smallest countable integer one, a clear indication that the empty set was part of an ordered number line.

In the current study, we explored if zero detectors reminiscent of real neurons would spontaneously emerge from a biologically inspired HCNN, i.e., a deep network that operates with receptive fields, lateral inhibition, and hierarchical processing layers to mimic the workings of the visual system deep learning network. Specifically, we explored if units tuned to the absence of stimuli as numerical value zero would emerge without explicit training. By silencing specific emerging units when testing the network's output, we probed the behavioral relevance of emerging units in solving a numerosity discrimination task. Such findings provide insight into the origin of numerical processes residing in the naive brain.

## Results

### Training and testing the deep hierarchical convolutional neural network

We trained a deep HCNN to perform only object classification in natural images. Like the brain's visual pathway, the model comprises several feedforward and retinotopically organized layers containing individual network units that mimic different types of visual neurons. Our previous work showed that robust numerosity representation reminiscent to that found in animal brains (Nieder 2016a) spontaneously emerged in an HCNN that was never explicitly trained on numerosity stimuli (Nasr et al., 2019).

**Figure 1:**
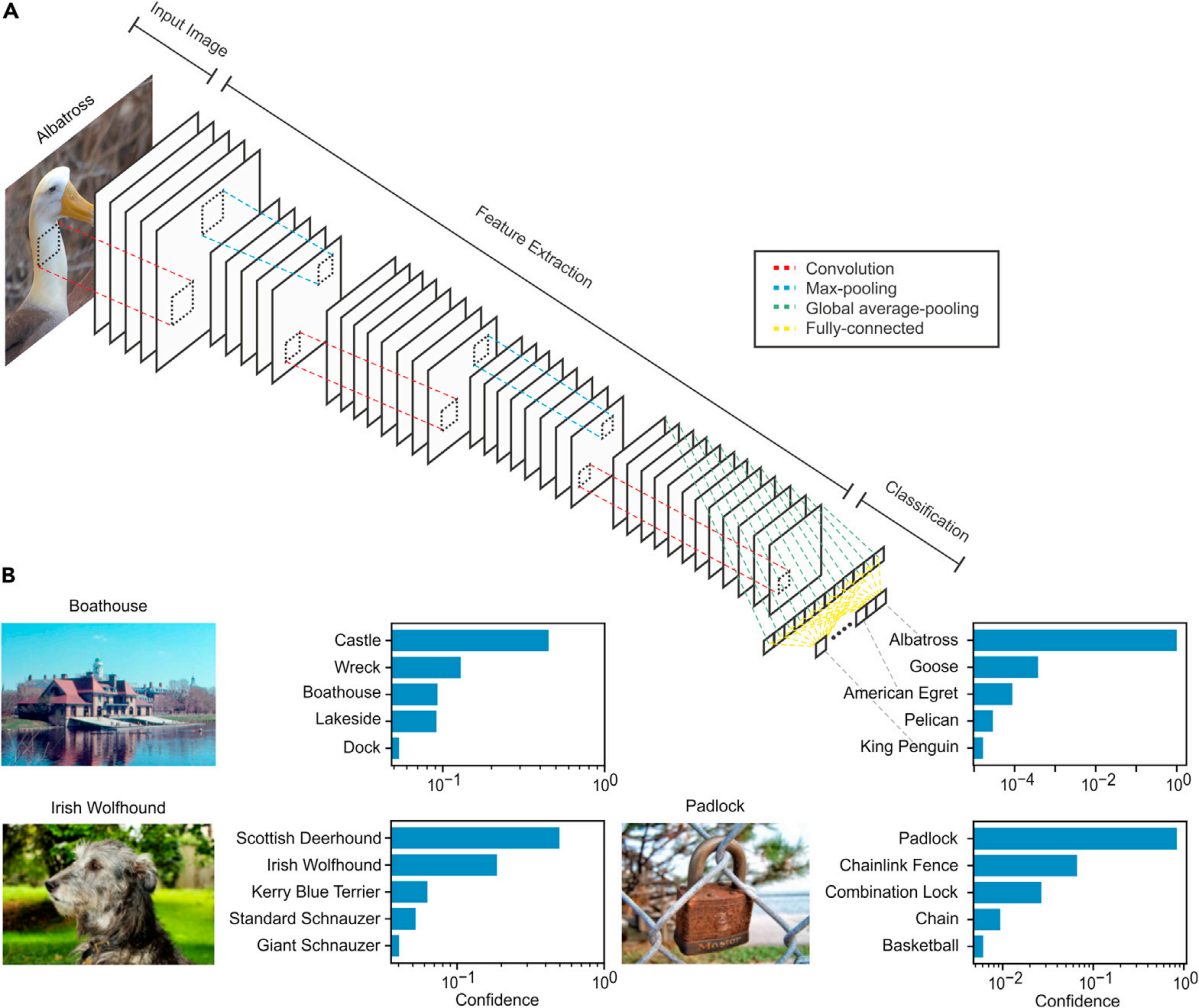
A hierarchical convolutional neural network (HCCN) for object recognition (A) Simplified network architecture. The network is divided into two parts: a feature extraction network and a classification network. The feature extraction network consists of convolutional layers that transform their inputs into multiple feature maps. Each feature map encodes the presence of some visual feature at each location in the input. Max-pooling layers introduce a degree of translational invariance by aggregating responses over small regions in their inputs. The classification network consists of a global average-pooling layer that computes the average response in each feature map and a fully connected layer where each unit encodes the probability that a certain object class is the most salient object in the input image. Right: top five predictions of the network ranked by confidence for the input image on the left depicting an albatross. (B) Further examples of images that were unseen by the network during training along with the top five predictions made by the network for each image. Ground-truth labels are shown above each image.

The network was subdivided into a *feature extraction* network that converts natural images into a high-level representation suitable for object categorization and a *classification* network that categorizes input images based on this high-level representation (Figure 1A; Table 1; see STAR Methods for details). To train the network on object recognition, we used the ILSVRC2012 ImageNet dataset, which contained 1.2 million images depicting objects of 1,000 categories (Krizhevsky et al., 2012). The network achieved a significant average classification accuracy of 48.6% (chance level = 0.1%; p < 0.001, binomial test) on a test set of new 50,000 images that were not used during training (Figure 1B).

**Table 1:** Description of the layers in the HCNN

Role	Layer	Type	Number of feature maps	Spatial size (pixels)	Kernel size (pixels)
Feature extraction	0	Input Image	3	224 × 224	–
	1	Convolutional	32	224 × 244	9 × 9
	2	Max-pooling	32	224 × 244	2 × 2
	3	Convolutional	48	112 × 112	9 × 9
	4	Max-pooling	48	112 × 112	2 × 2
	5	Convolutional	96	56 × 56	7 × 7
	6	Max-pooling	96	56 × 56	2 × 2
	7	Convolutional	192	28 × 28	5 × 5
	8	Max-pooling	192	28 × 28	2 × 2
	9	Convolutional	384	14 × 14	5 × 5
	10	Max-pooling	384	14 × 14	2 × 2
	11	Convolutional	768	7 × 7	5 × 5
	12	Convolutional	768	7 × 7	5 × 5
	13	Convolutional	768	7 × 7	5 × 5
Classification	14	Average-pooling	768	1 × 1	7 × 7
	15	Softmax classifier	1000	1 × 1	1 × 1

**Figure 2:**
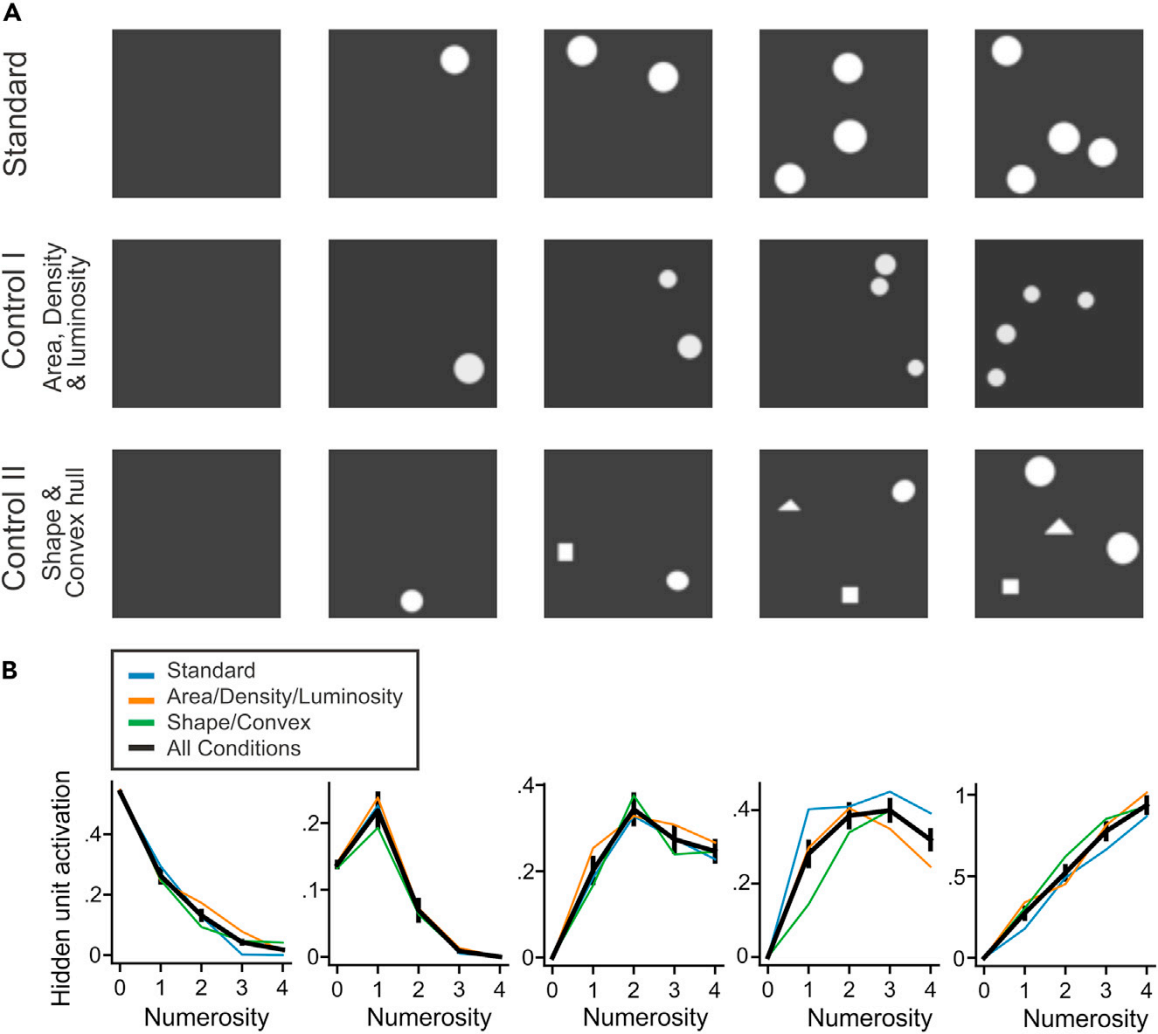
Network units spontaneously tuned to the empty set and countable numerosities (A) Samples from the stimulus sets used to study numerosity. Standard stimuli show dots that have a constant radius regardless of numerosity. Dots in control set 1 have constant total area, density, and luminosity across all numerosities. Dots in control set 2 have random shape and a triangular convex hull (for numerosities >2). (B) Tuning curves of individual numerosity-selective network units. From left to right, units were tuned to numerosity 0, 1, 2, 3, and 4. Colored curves represent average responses over each stimulus set. Black curves represent averages over all stimulus sets. Error bars indicate standard error measure.

We next investigated whether representation of the empty set (i.e., numerosity zero) has spontaneously emerged in the network after training only on natural object stimuli. To this aim, we presented the feature extraction network with newly generated images containing either a dark background representing the empty set or 1 to 4 randomly scattered dots (Figure 2A). To control for the effects of potential low-level visual features on network unit responses, the stimuli were divided into a standard set in which dots had a constant radius (Figure 2A, top row) and two control sets. In the first control set, average luminosity, total dot area, and average dot density were kept constant over each image (Figure 2A, middle row). In the second control set, the convex hull of the dots was kept triangular when possible and the shape of each dot was randomly chosen from a set of geometric shapes (circle, rectangle, ellipse, triangle) (Figure 2A, bottom row). A total of 600 dot images were presented to the feature extraction network while recording the responses of network units in the final layer of the network.

### Network units selective to empty sets and countable numerosities

We found network units in the final feature extraction layer that modulated their activity as a function of varying numbers of dots in the displays and irrespective of the appearance of the dot displays (Figure 2B). To statistically test the network units' response modulation, and to detect numerosity-selective units, we conducted a two-way ANOVA with “numerosity” (numerosity 0–4) and “stimulus set” (standard, control I, and control II) as main factors. We searched for network units that were only significantly selective for “numerosity,” but showed no significant effects for factor “stimulus set” or interactions (p < 0.01). This approach is identical to the way of identifying numerosity-selective neurons in the body of electrophysiological studies (Nieder, 2016a).

**Figure 3:**
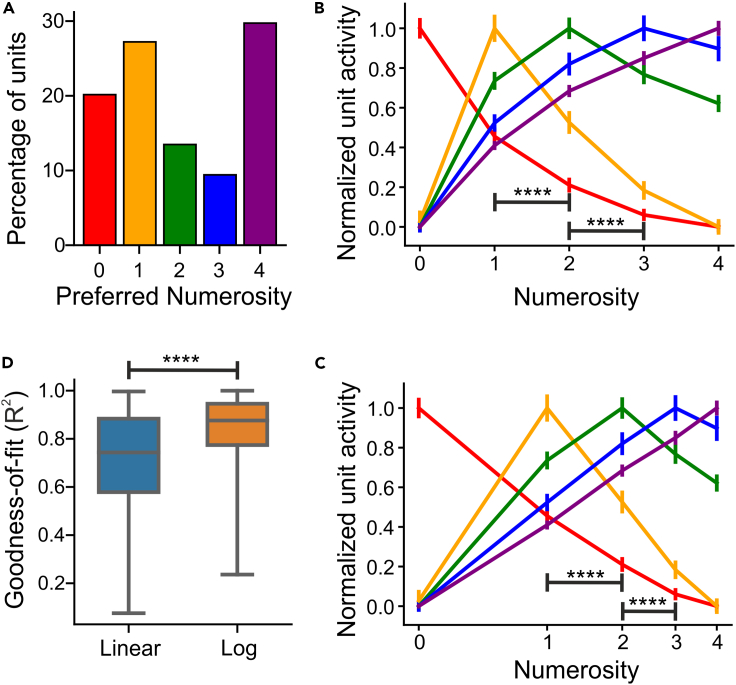
Network population coding of numerosity including zero (A) Distribution of preferred numerosities of network units. (B) Average tuning curves of network units for each preferred numerosity plotted on a linear scale. Error bars indicate standard error measure. Significance stars indicate p < 0.0001 with a Wilcoxon signed-rank test comparing responses of zero-preferring units to numerosity 1 versus 2 and 2 versus 3. (C) Same as (A) but plotted on a logarithmic scale. (D) Goodness-of-fit (R-Squared) measure for fitting the tuning curves of zero-tuned units with a straight line on linear versus logarithmic scale. Significance stars indicate p < 0.0001 with a Wilcoxon signed-rank test. Error bars indicate standard error measure.

Out of the 37,632 network units in the final layer of the feature extraction network, 3,939 network units (10.47%) were found to be numerosity selective. As previously reported, we found network units that were tuned to countable numerosity, i.e., they responded with maximum activity to one, two, three, or four items and showed a progressive decrease in activity as the presented numerosity deviated from the preferred numerosity (Figure 2B). In addition, we newly detected network units that were tuned to the empty set; such neurons showed maximum activity whenever the displays contained no items. Importantly, empty-set network units also exhibited a progressive decay in activity with increasing numerosity relative to the empty set. Empty-set network units responded more strongly to one item than to two items, thus exhibiting a numerical distance effect characteristic for numerical values ordered along a numerical continuum. The numerosity tuning functions of the network units turned out to be virtually identical to real neurons we had recorded previously. The network units' numerosity code was analogous to the tuning patterns of numerosity-selective neurons in monkeys (Nieder et al., 2002; Viswanathan and Nieder, 2013) and crows (Ditz and Nieder, 2015; Wagener et al., 2018), including the code for the empty set in both species. In addition, 20% of the network numerosity units preferred the empty set (Figure 3A), a frequency analogous to that observed in real neurons in monkeys (Ramirez-Cardenas et al., 2016) and crows (Kirschhock et al., 2021). Collectively, the tuned network units thus covered the entire range of presented numerosities.

To examine the population-level representation, we constructed population tuning curves by averaging the responses of network units that had the same preferred numerosity and normalizing them to the 0–1 range. The numerical distance effect suggested by the tuning of individual units was also clearly present on the population level (Figure 3B). Crucially, and analogous to the real neurons' empty-set neurons (Ramirez-Cardenas et al., 2016; Kirschhock et al., 2021), the activation of zero-preferring neurons was significantly higher for numerosity 1 compared with 2 (p < 0.0001; Wilcoxon signed-rank test) and for numerosity 2 compared with 3 (p < 0.0001; Wilcoxon signed-rank test) (Figure 3B). This indicates that the numerical distance effect was also present in the network units' representation of the empty set. Moreover, the network units' tuning curves systematically broadened with an increase in the preferred numerosity from 0–4 (Figure 3B). This was evidenced by the significant positive correlation between the preferred numerosity of each unit and the standard deviation of the Gaussian fit to its tuning curve (r = 0.56; p < 0.0001; Pearson correlation coefficient). This effect is also well known from real neurons recorded in humans (Kutter et al., 2018), monkeys (Nieder and Miller, 2003), and crows (Ditz and Nieder, 2016) and constituted a neuronal characteristic giving rise to the numerical size, i.e., a progressive difficulty to discriminate numerosity of constant absolute distance.

Furthermore, the resulting tuning curves exhibited clear asymmetric peak functions with more sharply decaying slopes toward smaller than larger numerosity when plotted on a linear number scale. However, just as for real monkey and crow neurons (Nieder and Miller, 2003; Ditz and Nieder, 2015), the peak functions became symmetric when plotted on a logarithmic scale (Figure 3C). Specifically, the tuning curves were better fit by symmetric Gaussian functions when plotted on logarithmic scale than when plotted on a linear scale (linear scale: r^2^ = 0.84; log scale: r^2^ = 0.9; p < 0.0001; Wilcoxon signed-rank test). Furthermore, we found that the tuning curves of the zero-preferring units were better fit with a straight line on a logarithmic scale than on a linear scale (Figure 3D). This suggested that, in consistence with the presence of a size effect, the tuning of zero-preferring units is logarithmic in nature. This non-linear, logarithmic compression of the neuronal number line is in agreement with the Weber-Fechner law that states that subjective differences between numerical values scale with the logarithm of physical numerical differences (Nieder 2020).

Although we focus on the low numerosity range (0–4) in the present study, previous studies have shown that numerosity representation in monkeys and HCNN models extends to cover a wider range of numerosities. To examine whether our findings concerning the empty set still held when network units were evaluated over a wider range of numerosity, we presented the network with numerosity stimuli covering the range from 0 to 30 (in steps of two numerosities). Consistently with our findings for the smaller (0–4) numerosity range, we found a significant and similar proportion (9.3%; 3,496/37,632) of network units in the final hidden layer to be selective to numerosity. In addition, we found a similar proportion (23.1%) being tuned to zero. The distribution of preferred numerosity (Figure S1A) exhibited a similar pattern under the two numerosity ranges, with similar proportions of units preferring low numerosity (0–3) and similar total proportions of units preferring numerosities ≥4 (29.7% versus 27.1% for the narrow and wide ranges, respectively). Additionally, the tuning curves of numerosity-selective units (Figure S1B) exhibited a similar pattern across the two ranges, indicating that our findings remained consistent when a wide range of numerosities is used.

To explore whether zero-tuned units would also emerge in the absence of any training (Kim et al., 2021), we analyzed the responses of units in the final hidden layer of our network model to numerosity stimuli before any training was performed. We found a significant portion (16.91%; 6,364/37,632) of numerosity-selective units in the untrained network (Figure S2A), a large proportion (32.6%) of which was tuned to the empty set. Similar to the findings of Kim et al. (2021), the distribution of preferred numerosity in the untrained was qualitatively skewed toward the extremities (Figure S2A). This indicated a bias toward monotonic units and was in contrast with the distribution of preferred numerosity in the trained network (Figure 3A) in which mid-range numerosities are more equally distributed. Although the tuning pattern of numerosity-selective units in the untrained network (Figure S2B) was similar to that in the network trained on object discrimination (Figure 3B), we observed significantly more variability in the average responses of numerosity-selective units in the untrained network compared with the trained network (average standard error in normalized response = 0.35 versus 0.043; p < 0.0001; Wilcoxon signed-rank test).

### “Behavioral output” based on the network's units

**Figure 4:**
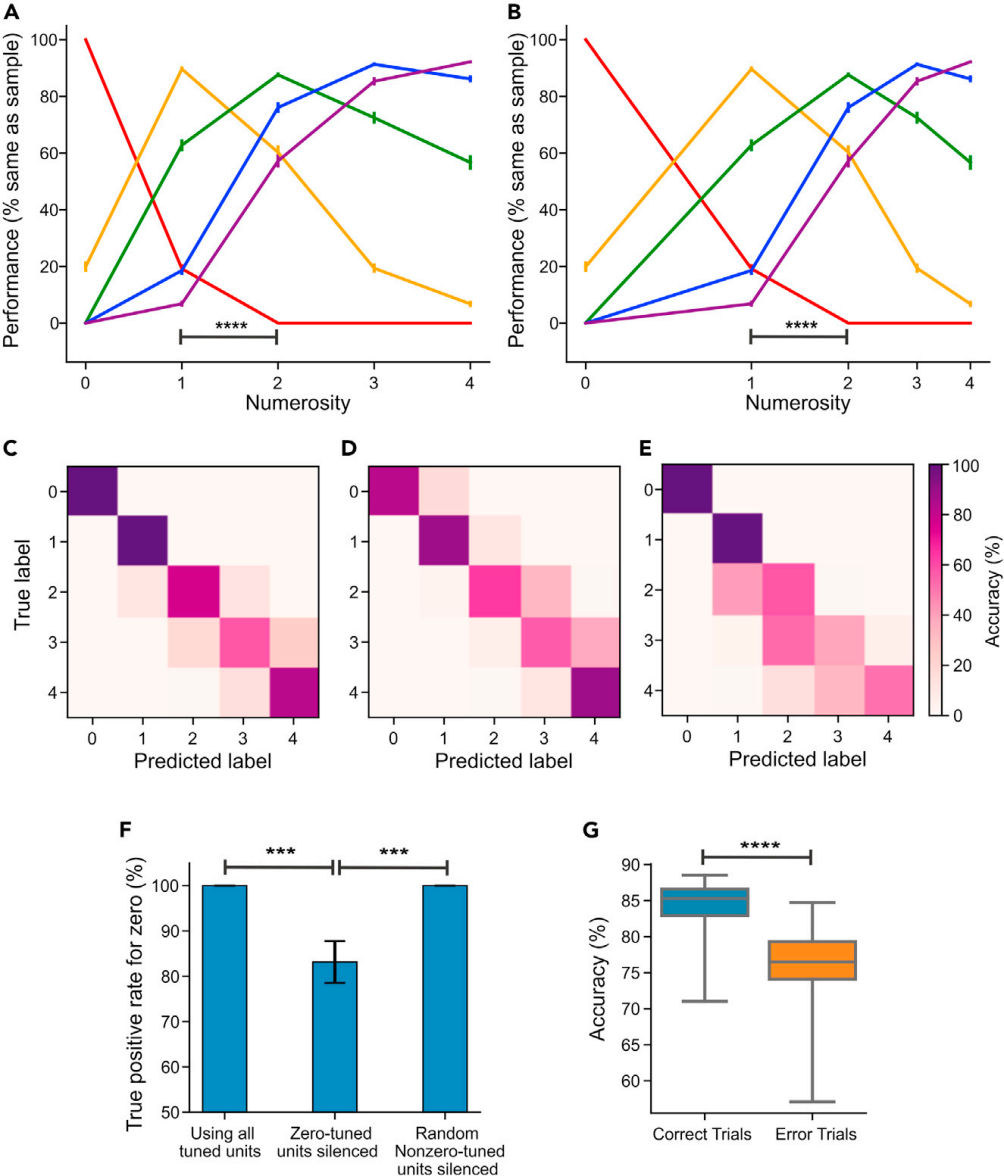
Network performance in numerosity matching and categorization (A) Behavioral performance functions of the network on the matching task plotted on a linear scale. Each curve shows the probability of predicting that the sample image contains the same number of items as the test image (peak of the function). Curves are calculated as averages over 50 repetitions of stimulus data generation, model training, and testing. Error bars indicate standard error measure over repetitions. Significance stars indicate a Wilcoxon signed-rank test (p < 0.0001) comparing responses of zero-preferring units to numerosity 1 versus 2. (B) Same as (A) but plotted on a logarithmic scale. (C) Confusion matrix of the network computed using the correct trials of the matching task. (D) Same as (C), but with zero-tuned units silenced. (E) Same as (C), but with randomly chosen nonzero-tuned units silenced. (F) True-positive rate for recognizing zero under different unit inclusion or exclusion conditions. Significance stars indicate p < 0.001 with a Wilcoxon signed-rank test. Error bars indicate standard error measure. (G) Numerosity categorization accuracy of the network computed separately for correct and error trials of the matching task. Significance stars indicate a Wilcoxon signed-rank test (p < 0.0001) comparing accuracies for correct and error triels. Error bars indicate standard error measure.

We then investigated whether the numerosity representation including zero developed by the network could be used to solve a task that requires the abstraction of absolute numerosity from low-level visual features. To this aim, we employed a numerosity matching task similar to those developed for monkeys, crows, and humans (Merten and Nieder, 2009; Ditz and Nieder, 2016). In every trial, the network was presented with two images each containing 0–4 dots, and a classifier was trained to use the activity of numerosity-selective network units to judge whether the two images contained the same number of dots. The network achieved an 80% accuracy on average (chance level was 50%) on test sets of image pairs that were not used during training. Accuracy for trials involving zero was even 96.6% (Figure 4A). To ensure that the network was not relying on low-level visual features to achieve this performance, we tested the network again only on stimuli from control sets I (area/density/luminosity) and II (shape/convex hull). Matching accuracy remained practically unchanged when tested on the two control sets (Control set I: 79.3%; Control set II: 80.3%). Accuracy for trials involving zero was also excellent (Control set I: 98.9%; Control set II: 93%). This showed that the numerosity-selective units can support reliable numerosity discrimination that does not rely on low-level visual features and that generalizes to the empty set.

We examined the detailed performance of the network (i.e., the “behavioral output” resulting from the working of the network units) as a function of presented numerosities. This clarified whether the network exhibited the numerical distance and size effects that characterize human and animal performance on similar tasks (Merten and Nieder, 2009), and whether these effects accommodate the empty set as an extension of the number line. As expected based on the numerical distance effect, we found that the network made more errors when distance between the presented numerosities were small than when the distance was large (Figure 4A). Crucially, the distance effect was also present when the network was judging empty sets: the network mistakenly matched zero to numerosity 1 more frequently than numerosity 2 (p < 0.0001; Wilcoxon signed-rank test). This indicated that the network accommodated the empty set as a numerical quantity on the number line.

Furthermore, we observed that the network's output became more imprecise with increasing numerical magnitudes, which resulted in the width of performance tuning functions to increase with numerosity (Figure 4A). To quantify this, we fit a Gaussian function to each performance tuning function and found a significant positive correlation between the standard deviation of the Gaussian fits and numerosity (r = 0.73; p < 0.0001; Pearson correlation coefficient). This indicated the presence of a size effect where discrimination accuracy worsened with increasing numerical magnitude as the network had more difficulty comparing large numerosities of a given numerical distance than small numerosities with the same distance. Analogously to the tuning curves of numerosity-selective units, the performance functions were better fit with symmetric Gaussian functions when plotted on a logarithmic scale than when plotted on a linear scale (linear scale: r^2^ = 0.84; log scale: r^2^ = 0.94; p < 0.0001; Wilcoxon signed-rank test). This indicated that, as expected by the Weber-Fechner law, the network's performance functions were asymmetric on a linear number line, but became more symmetric when plotted on a logarithmic scale (Figure 4B).

To further explore the network's numerical discrimination performance for the empty set and small numerosities, we trained a classifier to categorize dot patterns based on numerosity using the activity of numerosity-selective network units. The classifier was trained on images from the correct trials of the matching task and had an average classification accuracy of 84.6% (chance level was 20% for five numerosities) when tested on previously unseen images from other correct trials (Figure 4G). The confusion matrix of the classifier is shown in Figure 4C. Numerosity was accurately predicted by the classifier, which resulted in a high-accuracy diagonal. As a reflection of the numerical size effect, performance was high for low numerosity, particularly the empty set, and worsened as numerosity increased. As a reflection of the behavioral relevance of the tuned units, we tested the classifier for with error trials and found a significant decrease in accuracy (Figure 4G).

To specifically examine the behavioral relevance of zero-tuned units, we tested the classifier again while silencing either all zero-tuned units or an equal number of randomly chosen nonzero-tuned units as a control condition. We observed a significant decrease in the ability to recognize the empty set when zero-tuned units were silenced (p < 0.0001; Wilcoxon signed-rank test), both in the confusion matrix (Figure 4D) and the average accuracies (Figure 4F). In contrast, when an equal number of nonzero-tuned units were silenced, the ability to recognize zero remained completely unaffected in the confusion matrix (Figures 4E and 4F) and the average accuracy values (Figure 4F). As expected, accuracy of classifying countable numerosities was selectively impaired when nonzero-tuned units were silenced (Figure 4E). Moreover, silencing all units tuned to each countable numerosity led to a significant decrease in classification performance for that numerosity (p < 0.0001; Wilcoxon signed-rank test). These results indicated that zero-tuned units were specifically informative for recognizing and classifying the empty set.

## Discussion

The current study demonstrates that representations of “nothing” as numerosity zero spontaneously emerge in a deep learning neural network without any number training. The implemented HCNN showed architecture and function that closely mimicked the visual system (Nasr et al., 2019). Simply trained to classify images unrelated to numerical quantity, the network developed units tuned not only to countable numerosities but also to empty sets. These network units allowed for reliable and biologically realistic categorization of the number of items in dot displays controlled for non-numerical parameters. This demonstrates that deep-learning networks exhibit a surprising level of feature abstraction that may help to understand generalization processes in the brain.

### Empty-set network units represent numerosity zero as the smallest numerical value

About 10% of the network units in the topmost layers of our model spontaneously developed numerosity selectivity, either to numerosity zero or to countable numerosities. This proportion was comparable to findings in the brains of numerically-naive animals in which also around 10% of the neurons turned out to be numerosity selective (Viswanathan and Nieder, 2013; Wagener et al., 2018). The network units showed all the characteristics of real number neurons in agreement with the Weber-Fechner law, such as tuning to preferred numerosities, numerical distance and size effects, and logarithmic coding that have been found in crows, monkeys, and humans (Nieder and Miller, 2003; Merten and Nieder, 2009; Ditz and Nieder, 2015; Viswanathan and Nieder, 2015; Kutter et al., 2018). The same signatures have been reported using blood-oxygen-level-dependent activity in the human cerebral cortex (Piazza et al., 2004; Nieder, 2004; Jacob and Nieder, 2009; Kersey and Cantlon, 2017; Hofstetter et al., 2021; Cai et al., 2021). This validates the current model in terms of biological plausibility.

Of those numerosity-selective network units, 20% were tuned to the empty set. This proportion was again comparable to recordings in monkeys and crows (Ramirez-Cardenas et al., 2016; Kirschhock et al., 2021). Importantly, the activity of empty-set network units was relevant for the discrimination performance of the network: the exclusion of those units caused a significant decrease in the network's judgment of numerosity zero. Remarkably, empty-set network units showed maximum activity to numerosity zero and a progressive decrease in activity with increasing countable numerosities. This numerical distance effect in the network units' activity is a clear signature of the empty set as a numerical quantity representation (Merritt and Brannon, 2013; Nieder 2016b). This indicates that the network and its units ordered numerosity zero as the smallest numerical value along the number line.

### Numerosity coding in trained versus untrained networks

Although we explored the numerosity-representation capacity of a DNN that was only trained to perform object classification in natural images (Nasr et al., 2019), a recent computational study has shown that numerosity-selective units can emerge spontaneously in a completely untrained DNN (Kim et al., 2021). To explore whether zero-tuned units would also emerge in the absence of any training, we analyzed the responses of units in the final hidden layer of our network model to numerosity stimuli before any training was performed. Comparable to previous findings (Kim et al., 2021), we observed a significant portion of numerosity-selective units in the network that was entirely untrained. As a new finding, this also included a third of those units that were tuned to the empty set. Similar to previous findings (Kim et al., 2021), the distribution of preferred numerosity in our untrained DNN was qualitatively skewed toward the extremities. This indicates a bias toward monotonic units and was in contrast to the distribution of peak-tuned units in our network trained on object classification (Nasr et al., 2019). This finding in untrained DNN also contrasts with real neurons (Nieder, 2016a; Ramirez-Cardenas et al., 2016; Kirschhock et al., 2021) in which mid-range numerosities were represented in a more balanced way. In addition, we observed significantly more variability in the average responses of numerosity-selective units in the untrained network compared with the trained network. Taken together, these findings suggested that numerosity representations including the empty set are present even in entirely untrained network; however, such numerosity representations are more pronounced and mature, and thus more biologically relevant, in the network trained on object discrimination.

The spontaneous emergence of empty-set representation in a DNN that was not trained on any number-related task (or was even completely untrained) suggests a general mechanism for numerosity representation that is inherent to the hierarchical processing of visual stimuli and that extends to include the empty set. Recent studies show that convolutional neural networks (CNNs) that have been trained on visual tasks or have even remained completely untrained can be successful in unrelated visual tasks (Rosenfeld and Tsotsos, 2019; Razavian et al., 2014; Ulyanov et al., 2020). This phenomenon, referred to as “transfer learning” (Yosinski et al., 2014), could be part of the mechanism underlying previous findings concerning countable numerosity and our current findings concerning the empty set. Hierarchical processing of visual stimuli in a CNN automatically produces features that are useful for various visual tasks, and our findings indicate that empty-set representation, in addition to countable numerosity, is part of these emerging features.

### Zero capitalizes on already existing brain networks

We conclude that processing visual information—as simulated by a neuronal network mimicking the visual pathway—is sufficient to give rise to a visual number sense that includes empty sets as numerical quantity. The mere exposure to visual stimuli serves the emergence of empty-set units before any numerosity training. This may explain why some animals trained to order countable numerosities can immediately transfer their conceptual quantity knowledge to the empty set. Bees trained to order small numerosities according to a “less than” rule in transfer tests were spontaneously able to judge displays containing no item as numerically smaller than displays containing one or two items (Howard et al., 2018). This behavior suggests that neurons in the bee brain represented empty sets as numerical quantity before explicit training with empty sets.

Conceiving of “nothing” as a numerical quantity may in fact capitalize on already existing brain networks for visual processing. This may explain why neurons in the telencephalon of monkeys and crows represent numerosity zero (Ramirez-Cardenas et al., 2016; Kirschhock et al., 2021), and why cognitively advanced nonhuman animals with whom we share a nonverbal number system exhibit rudiments of a grasp of numerosity zero (Merritt et al. (2009), Kirschhock et al., 2021; Howard et al., 2018).

This, of course, is not to say that conceptions of numerical value zero would not be greatly improved and fully developed with learning and experience. This is particularly true for humans, the only species capable of a truly symbolic number representation. After it took humankind a surprisingly long time in history to appreciate zero as a number (Boyer, 1944), human culture now provides the learning background to conceive of the symbolic number zero (Nieder, 2016b). Even with this cultural background, the grasp of nothing as a quantity and later as a number is mentally demanding, as indicated by the protracted understanding of zero in child development (Wellman and Miller, 1986; Merritt and Brannon, 2013). Clearly, without zero becoming part of a combinatorial number symbol system, number theory and mathematics would be incomplete. However, despite the importance of culture, the biological primitives for understanding zero seem to be deeply rooted in the brains of phylogenetically diverse species.

### Limitations of the study

The current study focuses on the emergence on zero-tuned units in a CNN trained on natural stimuli in a task unrelated to numerosity. Although the study also provides preliminary results on the presence of zero-tuned units in the completely untrained network, albeit with a seemingly immature numerosity representation, it does not address the question of how numerosity representation including the empty set develops during training. Future work that examines the development of this representation as the network is being trained on natural stimuli (i.e., not just before and after training) could shed light on developmental mechanisms underlying numerosity representation in the brain.

## STAR★Methods

### Key resources table

**Table:** 

REAGENT or RESOURCE	SOURCE	IDENTIFIER
**Software and Algorithms**		

Python 3.8.5	Python Software Foundation	https://www.python.org/
Numpy 1.20.2	Open-source community project	https://numpy.org/
SciPy 1.5.0	Open-source community project	https://www.scipy.org/
Matplotlib 3.3.2	Michael Droettboom et al.	https://matplotlib.org/
PyTorch 1.8.1	Facebook's AI Research lab (FAIR)	https://pytorch.org/
Python code	Python Software Foundation	https://github.com/khalednasr/nn-numerosity-zero

### Resource availability

#### Lead contact

Further information and reasonable requests for resources and reagents should be directed to and will be fulfilled by the lead contact, Andreas Nieder (andreas.nieder@uni-tuebingen.de).

#### Materials availability

This study did not generate new unique reagents.

### Method details

#### Neural network model

We used a hierarchical convolutional neural network (HCCN; LeCun and Bengio, 1998) that was also designed and trained in our previous work (Nasr et al., 2019). The network was subdivided into a *feature extraction* network that converted natural images into a high-level representation suitable for object categorization, and a *classification* network that categorized input images based on this high-level representation (Figure 1A). The feature extraction network consisted of 8 convolutional layers and 5 pooling layers (Table 1). Network units in a convolutional layer performed local filtering operations analogous to those of simple cells in early visual cortex by computing a weighted sum of their inputs. The output of each unit in convolutional layers was normalized to have zero mean and unit standard deviation (*batch normalization*; Ioffe and Szegedy, 2015) and passed through a rectified-linear activation function f(x)=max(x,0). Units in pooling layers aggregated local responses similarly to complex cells by computing a maximum over local non-overlapping patches of size 2x2 pixels in their input, providing a degree of translational invariance and dimensionality reduction. In each layer, network units were organized topographically into multiple feature maps, with each feature map detecting the presence of a particular visual feature at all spatial locations in its input. As all units within the same feature map shared weights, each feature map was computing a convolution between its inputs a weight kernel. The weight kernels were initialized randomly and adapted through training to maximize the network’s performance on an object classification task. A simple form of lateral inhibition between network units that shared the same receptive field was implemented to diversify local responses. This was performed using the local response normalization function introduced by (Krizhevsky et al., 2012):

$$b_{x,y}^{i}=a_{x,y}^{i}/\left(k+\alpha \sum_{\text{max}(0,i-n/2)}^{\text{min}(N-1,i+n/2)}{\left(a_{x,y}^{j}\right)}^{2}\right)$$

where $a_{x,y}^{i}$ is the un-normalized response for the network unit at location x,y in the $i^{th}$ feature map, N is the total number of feature maps in the layer, and $b_{x,y}^{i}$ is the normalized response. The remaining variables were constants set to the values k=2, $\alpha =10^{-4}$, β=0.75, and n=15, which were based on the values used in (Krizhevsky et al., 2012). This enforced competition between network units by dividing the activity of each unit by a measure of the total activity of n units in adjacent feature maps, mimicking the effect of lateral inhibition. The classification network consisted of a global spatial averaging layer that computed the average response over all spatial locations in each of the final feature maps produced by the feature extraction network. This was followed by an output layer containing 1000 units, one per object category. The activity of each unit in that layer represented the probability that the input image depicted an object belonging to the corresponding category. Responses of units in the output layer were normalized using a *softmax* function $f\left(x\right)=e^{x_{i}}/\sum_{j}e^{x_{j}}$, where $x_{i}$ is the response of the $i^{th}$ network unit in the layer, to ensure that they represent a valid probability distribution. The initial weights of the network were randomly drawn from a uniform distribution (Xavier initialization; Glorot and Bengio, 2010) and optimized by minimizing the cross-entropy between the predicted object category probabilities and the ground-truth labels. The model was optimized for 10 epochs (complete presentations of the training data) using mini-batch gradient descent (Ruder, 2016; batch size = 256, learning rate = 0.1, momentum = 0.9) using the PyTorch framework (Paszke et al., 2017).

#### Stimuli

As in our previous work (Nasr et al., 2019), the network was trained on the ILSVRC2012 ImageNet dataset (Russakovsky et al. 2015) which contained 1.2 million images depicting objects of 1000 categories. The object classification accuracy of the model was evaluated on 50,000 images that were not seen by the model during training.

Numerosity stimuli consisted of 600 randomly generated images of size 224x224 pixels containing 0 to 4 dots. The network was tested on a standard stimulus set and two control sets that controlled for non-numerical cues, with equal number of images for each numerosity and stimulus set combination. Stimuli for zero consisted of an image containing only a dark background. To introduce variation into the deterministic network’s response to this constant stimulus, we injected multiplicative Gaussian noise (μ=1.0,σ=0.15) into the outputs of all convolutional units. The introduction of noise did not impair the network’s overall object classification accuracy (48.6% with noise; 49.9% without noise). In the standard stimulus condition, average dot radius (18 pixels) was kept constant regardless of numerosity and dots were randomly scattered on the stimulus image. In control set 1, the total dot area in each image was fixed to 1200 pixels, the average distance between pairs of dots was kept in the 90-100 pixels range, and the average luminosity was kept constant at 20%. In control set 2, the shapes of individual dots were randomly sampled from a fixed set of shapes (circle, rectangle, ellipse, and triangle), and the overall convex hull of the dots in each image was fixed to a triangle (for numerosities larger than 2) of random location and orientation.

### Quantification and statistical analysis

#### Analysis of network units

Following training for object classification, the feature extraction network was presented with numerosity stimuli and the responses of units in the final layer were analyzed. Similar to the approach used to detect numerosity selectivity in our previous work (Nasr et al., 2019) and previous studies on monkey, crows, and humans (Nieder and Miller, 2003; Ditz and Nieder, 2016; Kutter et al., 2018), numerosity-selective units were found using a two-way ANOVA with numerosity (5 levels) and stimulus set (3 levels) as factors. Network units showing a significant effect for numerosity (P < 0.01) but no significant effect for stimulus set or interaction were labeled as numerosity-selective. Tuning curves for each unit were calculated by averaging the unit’s responses to all images depicting each numerosity. Preferred numerosity of each unit was defined as the numerosity that elicited the greatest average response, i.e., the peak of the unit’s tuning curve. Population tuning curves were calculated by averaging the responses of units that had the same preferred numerosity and normalizing them to the 0-1 range. Responses of units in the untrained network were analyzed in a similar manner, but the analysis results were averaged over 100 repetitions of network initialization with random weights, stimulus generation and presentation.

#### Numerosity matching and classification

To study the behavioral relevance of the numerosity-selective network units, simple classifiers were trained to solve a numerosity matching task using their activity. In each trial, the feature extraction network was presented with two newly generated images containing dot patterns, and the responses of numerosity-selective units were used as input to a support vector machine (SVM) classifier that was trained to judge whether the two images contained the same number of dots and tested on a set of previously unseen images. The classifier was trained on 600 trials and tested on another 600 trials. The data generation, training, and testing procedure was repeated 50 times and subsequent analysis (Figure 4) was conducted over those repetitions. Test trials were divided into correct and error trials based on the classifier’s performance. In each repetition, a multi-class one-vs-one SVM classifier was trained to categorize images based on numerosity using the activity of numerosity-selective units. The multi-class SVM was trained on half the images in the correct trials and tested either on the other half or on the error trials. To analyze behavior relevance, zero-tuned units (or a randomly chosen equal number of nonzero-tuned units in the control condition) were silenced by setting their activity to zero while the classifier was tested.

## Data Availability

• The data can be downloaded from the following repository: https://github.com/khalednasr/nn-numerosity-zero • The code scripts can be downloaded from the following repository: https://github.com/khalednasr/nn-numerosity-zero

## References

[bib1] Boyer C.B. (1944). Zero: the symbol, the concept, the number. Natl. Math. Mag..

[bib2] Cai Y., Hofstetter S., van Dijk J., Zuiderbaan W., van der Zwaag W., Harvey B.M., Dumoulin S.O. (2021). Topographic numerosity maps cover subitizing and estimation ranges. Nat. Commun..

[bib54] Castaldi E., Aagten-Murphy D., Tosetti M., Burr D., Morrone M.C. (2016). Effects of adaptation on numerosity decoding in the human brain. NeuroImage.

[bib3] Castaldi E., Burr D., Turi M., Binda P. (2020). Fast saccadic eye-movements in humans suggest that numerosity perception is automatic and direct. Proc. Biol. Sci..

[bib4] Ditz H.M., Nieder A. (2015). Neurons selective to the number of visual items in the corvid songbird endbrain. Proc. Natl. Acad. Sci. U S A.

[bib55] Ditz H.M., Nieder A. (2016). Sensory and working memory representations of small and large numerosities in the crow endbrain. J. Neurosci..

[bib5] Ditz H.M., Nieder A. (2020). Format-dependent and format-independent representation of sequential and simultaneous numerosity in the crow endbrain. Nat. Commun..

[bib6] Fornaciai M., Park J. (2018). Early numerosity encoding in visual cortex is not sufficient for the representation of numerical magnitude. J. Cogn. Neurosci..

[bib7] Glorot, X. & Bengio, Y. (2010). Understanding the difficulty of training deep feedforward neural networks. In: Proceedings of the Thirteenth International Conference on Artificial Intelligence And Statistics. (pp. 249-256). JMLR Workshop and Conference Proceedings.

[bib8] Hofstetter S., Cai Y., Harvey B.M., Dumoulin S.O. (2021). Topographic maps representing haptic numerosity reveals distinct sensory representations in supramodal networks. Nat. Commun..

[bib9] Howard S.R., Avarguès-Weber A., Garcia J.E., Greentree A.D., Dyer A.G. (2018). Numerical ordering of zero in honey bees. Science.

[bib10] Ioffe S., Szegedy C., Bach F., Blei D. (2015). International Conference on Machine Learning.

[bib11] Izard V., Sann C., Spelke E.S., Streri A. (2009). Newborn infants perceive abstract numbers. Proc. Natl. Acad. Sci. U S A.

[bib12] Jacob S.N., Nieder A. (2009). Tuning to non-symbolic proportions in the human frontoparietal cortex. Eur. J. Neurosci..

[bib13] Kersey A.J., Cantlon J.F. (2017). Neural tuning to numerosity relates to perceptual tuning in 3-6-year-old children. J. Neurosci..

[bib14] Kim G., Jang J., Baek S., Song M., Paik S.B. (2021). Visual number sense in untrained deep neural networks. Sci. Adv..

[bib15] Kirschhock M.E., Ditz H.M., Nieder A. (2021). Behavioral and neuronal representation of numerosity zero in the crow. J. Neurosci..

[bib16] Krizhevsky A., Sulskever I., Hinton G.E. (2012). ImageNet classification with deep convolutional neural networks. Adv. Neural Inf. Process. Syst..

[bib17] Kutter E.F., Bostroem J., Elger C.E., Mormann F., Nieder A. (2018). Single neurons in the human brain encode numbers. Neuron.

[bib18] LeCun Y., Bengio Y. (1998). Convolutional networks for images, speech, and time-series. Handb. Brain Theor. Neural Networks.

[bib19] Merritt D.J., Brannon E.M. (2013). Nothing to it: precursors to a zero concept in preschoolers. Behav. Process..

[bib20] Merritt D.J., Rugani R., Brannon E.M. (2009). Empty sets as part of the numerical continuum: conceptual precursors to the zero concept in rhesus monkeys. J. Exp. Psychol. Gener..

[bib21] Merten K., Nieder A. (2009). Compressed scaling of abstract numerosity representations in adult humans and monkeys. J. Cogn. Neurosci..

[bib22] Nasr K., Viswanathan P., Nieder A. (2019). Number detectors spontaneously emerge in a deep neural network designed for visual object recognition. Sci. Adv..

[bib23] Nieder A. (2016). The neuronal code for number. Nat. Rev. Neurosci..

[bib24] Nieder A. (2016). Representing something out of nothing: the dawning of zero. Trends Cogn. Sci..

[bib25] Nieder A. (2020). The adaptive value of numerical competence. Trends Ecol. Evol..

[bib26] Nieder A. (2004). The number domain- can we count on parietal cortex?. Neuron.

[bib27] Nieder A., Miller E.K. (2003). Coding of cognitive magnitude: compressed scaling of numerical information in the primate prefrontal cortex. Neuron.

[bib28] Nieder A., Freedman D.J., Miller E.K. (2002). Representation of the quantity of visual items in the primate prefrontal cortex. Science.

[bib29] Okuyama S., Kuki T., Mushiake H. (2015). Representation of the numerosity 'zero' in the parietal cortex of the monkey. Sci. Rep..

[bib30] Park J., DeWind N.K., Woldorff M.G., Brannon E.M. (2016). Rapid and direct encoding of numerosity in the visual stream. Cereb. Cortex.

[bib56] Paszke A., Gross S., Chintala S., Chanan G., Yang E., DeVito Z., Lin Z., Desmaison A., Antiga L., Lerer A. (2017). 31st Conference on Neural Information Processing Systems (NIPS 2017).

[bib32] Piazza M., Izard V., Pinel P., Le Bihan D., Dehaene S. (2004). Tuning curves for approximate numerosity in the human intraparietal sulcus. Neuron.

[bib33] Ramirez-Cardenas A., Nieder A. (2019). Working memory representation of empty sets in the primate parietal and prefrontal cortices. Cortex.

[bib34] Ramirez-Cardenas A., Moskaleva M., Nieder A. (2016). Neuronal representation of numerosity zero in the primate parieto-frontal number network. Curr. Biol..

[bib35] Razavian A.S., Azizpour H., Sullivan J., Carlsson S. (2014). Proceedings of the IEEE Conference on Computer Vision and Pattern Recognition Workshops.

[bib36] Rosenfeld A., Tsotsos J.K., Canadian Conference on Computer and Robot Vision (2019). 2019 16th Conference on Computer and Robot Vision (CRV).

[bib37] Ruder S. (2016). An overview of gradient descent optimization algorithms. arXiv.

[bib38] Rugani R., Regolin L., Vallortigara G. (2008). Discrimination of small numerosities in young chicks. J. Exp. Psychol. Anim. Behav. Process..

[bib39] Russakovsky O., Deng J., Su H., Krause J., Satheesh S., Ma S., Fei-Fei L. (2015). Imagenet large scale visual recognition challenge. Int. J. Comput. Vis..

[bib51] Simonyan K., Zisserman A. (2015). Very deep convolutional networks for large-scale image recognition. CoRR.

[bib41] Stoianov I., Zorzi M. (2012). Emergence of a 'visual number sense' in hierarchical generative models. Nat. Neurosci..

[bib50] Ulyanov D., Vedaldi A., Lempitsky V. (2020). Deep image prior. Int. J. Comput. Vis..

[bib43] Viswanathan P., Nieder A. (2015). Differential impact of behavioral relevance on quantity coding in primate frontal and parietal neurons. Curr. Biol..

[bib44] Viswanathan P., Nieder A. (2013). Neuronal correlates of a visual "sense of number" in primate parietal and prefrontal cortices. Proc. Natl. Acad. Sci. U S A.

[bib45] Wagener L., Loconsole M., Ditz H.M., Nieder A. (2018). Neurons in the endbrain of numerically naive crows spontaneously encode visual numerosity. Curr. Biol..

[bib46] Wellman H.M., Miller K.F. (1986). Thinking about nothing: development of concepts of zero. Br. J. Dev. Psychol..

[bib47] Yamins D.L., DiCarlo J.J. (2016). Using goal-driven deep learning models to understand sensory cortex. Nat. Neurosci..

[bib48] Yamins D.L., Hong H., Cadieu C.F., Solomon E.A., Seibert D., DiCarlo J.J. (2014). Performance-optimized hierarchical models predict neural responses in higher visual cortex. Proc. Natl. Acad. Sci. U S A.

[bib53] Yosinski J., Clune J., Bengio Y., Lipson H. (2014).

