# Supplementary material for: Spontaneous representation of numerosity zero in a deep neural network for visual object recognition
Source: iScience. 2021 Oct 15;24(11):103301. doi: 10.1016/j.isci.2021.103301 (PMC8571726; doi:10.1016/j.isci.2021.103301)
Supplement: Document S1. Figures S1 and S2 [file mmc1.pdf]

iScience, Volume *24*

Supplemental information

Spontaneous representation of numerosity zero in a deep neural network for visual object recognition

Khaled Nasr and Andreas Nieder

Supplemental Information

Spontaneous representation of numerosity zero in a deep neural network for visual object recognition

Khaled Nasr and Andreas Nieder

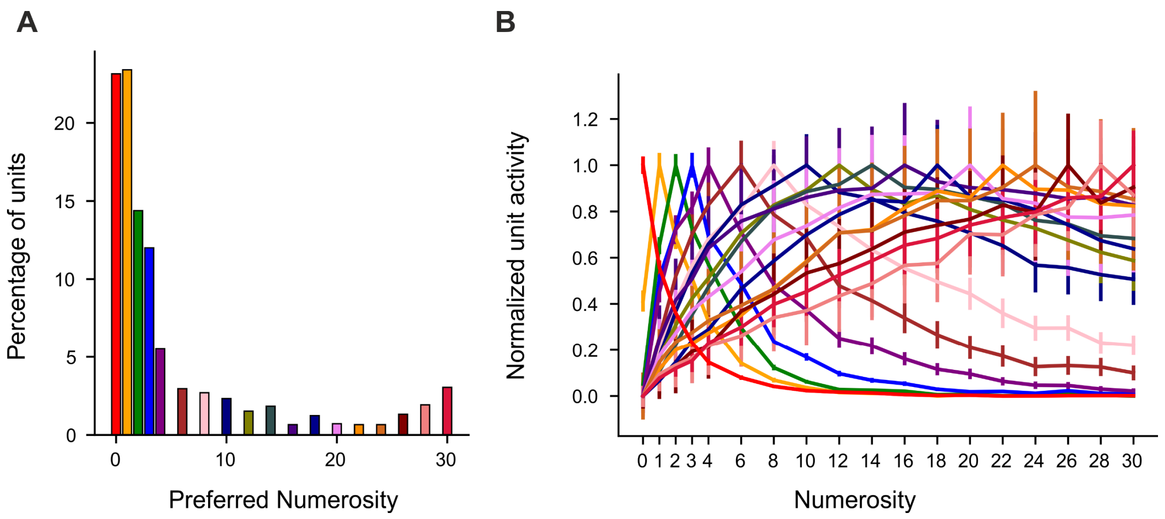

Fig. S1. Network population coding of a wider range of numerosity including zero, related to Figure 3.
(A) Distribution of preferred numerosities of network units for a wider (0-30) range of numerosity stimuli. **(B)** Average tuning curves of network units for each preferred numerosity plotted on a linear scale. Error bars indicate standard error measure.

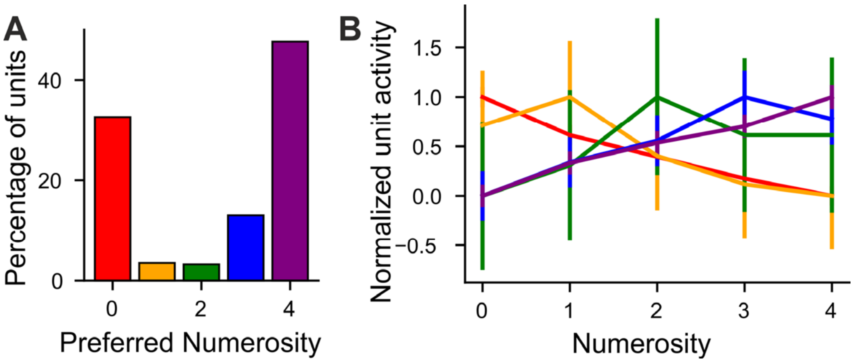

Fig. S2. Network population coding of numerosity in absence of training, related to Figure 3.
(A) Distribution of preferred numerosities of units in the untrained network. **(B)** Average tuning curves of network units for each preferred numerosity plotted on a linear scale. Error bars indicate standard error measure.
